# Association Between Inflammatory Mediators and Pulmonary Blood Flow in a Rabbit Model of Acute Pulmonary Embolism Combined With Shock

**DOI:** 10.3389/fphys.2020.01051

**Published:** 2020-09-02

**Authors:** Yuting Wang, Delong Yu, Yijun Yu, Xiaoyan Liu, Liqun Hu, Ye Gu

**Affiliations:** Department of Cardiology, Wuhan Fourth Hospital, Puai Hospital Affiliated to Tongji Medical College, Huazhong University of Science and Technology, Wuhan, China

**Keywords:** acute pulmonary embolism, pro-inflammatory cytokines, TNF-α, IL-1β, IL-6, pulmonary vasoconstriction, pulmonary blood flow attenuation

## Abstract

**Background:**

The pro-inflammatory cytokines were detected in pulmonary embolism (PE) and non-pulmonary embolism (non-PE) tissues to explore the role of inflammation responses and their relationship with the pulmonary blood flow in a rabbit model of acute pulmonary embolism combined with shock.

**Methods and Results:**

Nineteen rabbits were randomly divided into sham operation group (S group, *n* = 8) and massive PE (MPE group, *n* = 11). The MPE model was established by injecting the autologous blood clots into the main pulmonary artery of rabbit. Pulmonary angiography showed that the pulmonary circulation time was significantly prolonged in the MPE group, and pulmonary blood flow was attenuated at 120 min post PE. Hematoxylin–eosin (HE) staining revealed enhanced inflammatory cell infiltration around the pulmonary vessels in PE and non-PE tissues, and obvious edema on the perivascular region. Meanwhile, the expressions of inducible nitric oxide synthase (iNOS) and arginase 1 (Arg-1) in pulmonary vascular and alveolar tissues were significantly upregulated and the iNOS/Arg-1 ratio was significantly higher in the MPE group than in the S group. Moreover, the levels of tumor necrosis factor-alpha (TNF-α) and interleukin-1 beta (IL-1β) were also significantly increased in PE and non-PE tissues, and interleukin-6 (IL-6) level was significantly increased in non-PE tissues in the MPE group as compared to the S group. Thromboxane A2 (TXA_2_) and alpha smooth muscle actin (α-SMA) levels were significantly higher in both PE and non-PE tissues in the MPE group than in the S group.

**Conclusion:**

Activation of inflammation mediators in PE and non-PE tissues might be one of the crucial factors responsible for pulmonary vasculature constriction and pulmonary blood flow attenuation in this MPE model.

## Introduction

Blockade of pulmonary vasculature by the blood clots in the case of acute pulmonary embolism (APE) can lead to pulmonary hypertension and cardiopulmonary injury (Zhao et al., 2015; Wang et al., 2019). Loss of blood flow to the distal pulmonary vasculature and reduced blood filling to the left ventricle post APE might also result in systemic circulatory failure (shock) and death. Several studies have explored means to reduce the high mortality in the acute phase of massive PE (MPE) and improve its long-term prognosis, however, the achieved benefits were limited (Kinoshita et al., 2000; Neto-Neves et al., 2011; Chun et al., 2012). Our previous studies (Yu et al., 2018; Wang et al., 2019) found that reduced pulmonary artery blood flow in case of APE was jointly induced by pulmonary artery spasm in PE tissues and pulmonary vasculature spasm in non-PE regions. Nonetheless, the detailed mechanism regarding pulmonary vasospasm of PE and non-PE tissues in MPE is not fully understood. A previous study indicated that hypoxia-induced sympathetic system activation served as one of the determinants of pulmonary vasculature spasm and blood flow attenuation in the whole lung during MPE combined with shock (Chun et al., 2012). In the setting of PE, hypoxia is also an important driving factor to enhance inflammatory response, and hypoxia-induced generation of reactive oxygen free radicals is a known stimulator of inflammation (He et al., 2016; Chen et al., 2020). Hence, hypoxia might play a crucial role in PE-induced inflammatory response. In addition, PE-induced pulmonary vascular spasm could also result in hypoxia and lead to a vicious circle of hypoxia-inflammation cascade in both PE and non-PE tissues in this model. Thus far, the association between inflammatory response and pulmonary blood flow failure in MPE remains elusive. This study therefore explored the expressions of inflammatory mediators in PE and non-PE tissues and its relationship with the pulmonary blood flow change in a rabbit model of MPE combined with shock.

## Materials and Methods

### Animals

All animal treatment complied with the Guide for the Care and Use of Laboratory Animals published by the US National Institute of Health (NIH Publication No. 85-23, revised 1996), and the study protocol was approved by the Animal Care Committee at Huazhong University of Science and Technology. Healthy adult New Zealand rabbits, weighing 2.5–3.0 kg, were purchased from the Experimental Animal Center of Tongji Medical College, Huazhong University of Science and Technology. The rabbits were housed under standard conditions with free access to food and drinking water for 1 week before the experiments. Nineteen rabbits were used: 8 rabbits underwent sham operation (S group, puncture and catheterization, followed by bolus injection of saline), and 11 rabbits were used to establish the MPE model (puncture and catheterization, followed by bolus injection of autologous blood clots until shock status was reached).

### Anesthesia and Custody

All surgical procedures were performed under sodium pentobarbital anesthesia, in that 3% sodium pentobarbital (20 mg/kg) was injected through the rabbit ear vein. After anesthetic induction, the rabbit was fixed on the operating table in the supine position and heart rate and blood pressure were monitored by the cardiogram monitor (LEAD-7000).

### Paracentesis, Catheterization, and the Establishment of MPE Model Combined With Shock

First, skin in the right groin area was prepared and disinfected. After isolating the femoral artery and femoral vein, they were punctured using the Seldinger puncturing method. A 4F Cordis catheter (Cordis Corporation, Florida, United States) was placed in the main trunk of the pulmonary artery through the femoral vein under radiographic guidance. The 5F sheath tube (Radiofocus TERUMO) was inserted into the femoral artery. Second, the MPE model was established as previously described (Yu et al., 2018; Wang et al., 2019). The autologous blood clots [3 mm (± 0.5) × 10 mm] were prepared and placed at the tail end of the 4F catheter and then slowly injected into the main pulmonary artery of the rabbit with 5 mL saline. Autologous blood clots were delivered to the main pulmonary artery in a two-stage manner. In the first stage, a single clot was injected at 60-s intervals four times. Then, the tail end of the 4F Cordis catheter was connected to the pressure transducer for real-time continuous monitoring of the mean arterial pressure (MAP) and mean pulmonary artery pressure (MPAP) by LEAD-7000 monitor (Sichuan Jinjiang Electronic Technology Co., China). The model was considered successful when both following criteria were reached for 2 min: (a) The MAP reduced to < 60 mmHg, and (b) the MPAP increased to up to 2 times the baseline level. MAP and MPAP were dynamically monitored during this period. Third, if MAP and MPAP did not reach the defined levels after the four times of clot injections after the first stage (within 15 min after the first clot injection), more clots were injected at 60-s intervals at the second stage. All rabbits reached the shock status with a maximal of 3 additional clot injections in this experiment. MAP and MPAP were dynamically monitored before PE, during the PE procedure, at the moment of shock status post bolus injection of autologous blood clots, and at 120 min after initiation of MPE. MPE with shock was achieved in all rabbits within 15 min after the clot injection.

### Pulmonary Artery Angiography

Pulmonary arterial blood flow imaging was obtained by pulmonary artery angiography (Allura Xper FD20 Philips). Pulmonary embolism, pulmonary blood flow imaging characteristics, and pulmonary circulation times were compared between the two groups. The pulmonary circulation time was determined by calculating the time taken for the contrast medium to reach from the initiation site of the pulmonary artery trunk to the left atrium through the pulmonary artery trunk and branches, pulmonary capillary, and pulmonary vein. The pulmonary circulation time was calculated for all of the survived rabbits at 120 min, which was the designed study end point [S group (*n* = 8), MPE group (*n* = 6)].

### Histological Detection

At the end of the experiment, the rabbits were sacrificed under deep anesthesia (Yu et al., 2018). Based on a previous report (Dias-Junior et al., 2006), 120 min post MPE was chosen for measurement of inflammation. Tissues from four rabbits in the S group and five in the MPE group were randomly selected for pathological detection by hematoxylin–eosin (HE) and immunohistochemistry staining. Similarly, tissues from four and five rabbits in the S and MPE groups, respectively, were used for PCR and enzyme-linked immunosorbent assay (ELISA)-based experiments. Lung tissues were excised and underwent fixation with paraformaldehyde. PE and non-PE tissues were cut into sections for pathological examination after paraffin embedding, respectively. The HE stain was used to analyze the pathology of PE and non-PE tissues. Immunohistochemistry staining was also performed on lung tissue sections with the following antibodies: inducible nitric oxide synthase (iNOS) (1:100, abcam, Cambridge, MA, United States), arginase 1 (Arg-1) (1:100, abcam), and alpha smooth muscle actin (α-SMA) (1:100, abcam). The pathological changes were quantified using commercial software (Image-Pro Plus, Media Cybernetics, Inc., Rockville, Maryland, United States) and evaluated by assessing the percentage of the positive area. In addition, the levels of thromboxane A2 (TXA_2_) and prostaglandin I2 (PGI_2_) in PE and non-PE tissues were evaluated by ELISA.

### Determination of the mRNA Expression of Pro-inflammatory Cytokines in PE and Non-PE Tissues by Real-Time Polymerase Chain Reaction

Total RNA was extracted from PE and non-PE lung tissues using RNA Tissue Mini Kit (Takara, Dalian, Liaoning, China) according to the manufacturer’s instructions. Reverse transcription and cDNA synthesis were performed using PrimeScript RT Master Mix Perfect Real Time (Takara, Dalian, Liaoning, China). Real-time polymerase chain reaction was performed to detect the expression of various cytokines such as tumor necrosis factor-alpha (TNF-α), interleukin-1 beta (IL-1β), and interleukin-6 (IL-6) by QuantiFast SYBR Green PCR Kit (Qiagen, Dusseldorf, Germany) and Bio-Rad CFX96 (Bio-Rad, Hercules, CA, United States) according to the manufacturer’s instructions. PCR primers are shown in Table 1. Relative gene expression was calculated using the 2^–ΔΔCT^ method.

**TABLE 1:** PCR primers sequences.

Forward/reverse	Sequence (5′–3′)
β-Actin	S: 5′-CCCAGATCATGTTCGAGACGT-3′
	F: 5′-TAGCCCTCGTAGATGGGCAC-3′
IL-1β	S: 5′-TCCTGCGTGATGAAAGACGAT-3′
	F: 5′-GGAAGACGGGCATGTACTCTGT-3′
IL-6	S: 5′-CTACCGCTTTCCCCACTTCA-3′
	F 5′-GGCAGGTCTCATTATTCACCG-3′
TNF-α	S: 5′-AGTAGCAAACCCGCAAGTGG-3′
	F: 5′-CCTTGTTCGGGTAGGAGACG-3′

*IL-1β, interleukin-1 beta; IL-6, interleukin-6; TNF-α, tumor necrosis factor*-alpha.**

### Statistical Analysis

All statistical data were expressed as mean ± SD. All data were normally distributed as assessed by the Shapiro–Wilk test. Student’s *t*-test was employed to evaluate the differences between the two groups. *P* < 0.05 was considered to indicate statistical significance. All statistical analyses were performed with IBM SPSS 19.0 software (IBM, NY, United States).

## Results

All eight rabbits in the S group and six out of eleven rabbits in the MPE group survived at the end of the study. Five rabbits in the MPE group died at 7, 8, 21, 32, and 36 min after reaching the shock status, most likely because of irreversible shock.

### Pulmonary Angiography Assessment Results

**FIGURE 1:**
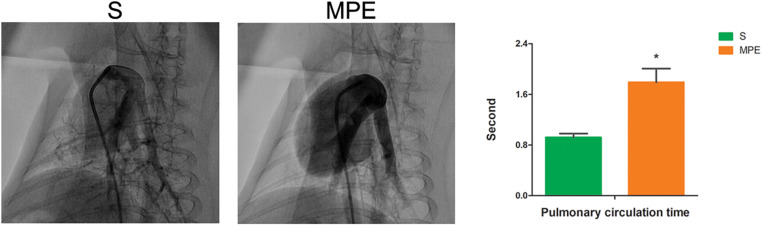
Pulmonary angiography results in S and MPE groups. The pulmonary circulation time was significantly prolonged in the MPE group (*n* = 6) compared with that of the S group (*n* = 8). *P < 0.05 vs. S group. S, sham operation group; MPE, massive pulmonary embolism group.

Pulmonary angiography was performed at 120 min post pulmonary occlusion to explore the features of pulmonary circulation. The perfusion of lung field was obviously decreased, and the pulmonary blood flow to the left and right lower lobes was attenuated in the MPE group. The pulmonary circulation time was significantly prolonged in the MPE group compared to the S group (Figure 1, *P* < 0.05).

### HE Pathology Results

**FIGURE 2:**
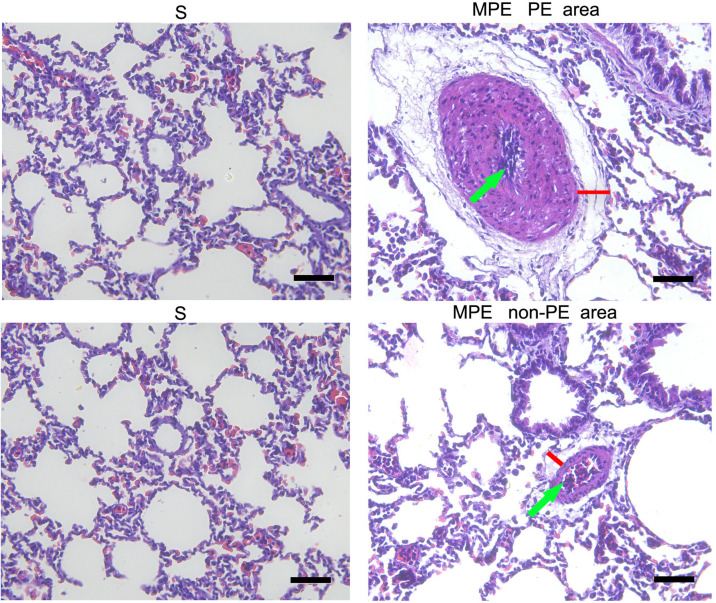
Histological features of PE and non-PE tissues with hematoxylin and eosin (HE). The interstitial edema (red lines) and the inflammatory cell infiltration (green arrows). PE, pulmonary embolism tissue; non-PE, non-pulmonary embolism tissue. S, sham operation group (*n* = 4); MPE, massive pulmonary embolism group (*n* = 5). Scale bars: 50 μm.

The morphological change of lung tissue is presented in Figure 2. Obvious interstitial edema (red lines) and enhanced inflammatory cell infiltration in the pulmonary vessels (green arrows) were seen in the pulmonary vasculature of both PE and non-PE tissues in the MPE group.

### iNOS and Arg-1 Expression in PE and Non-PE Tissues

**FIGURE 3:**
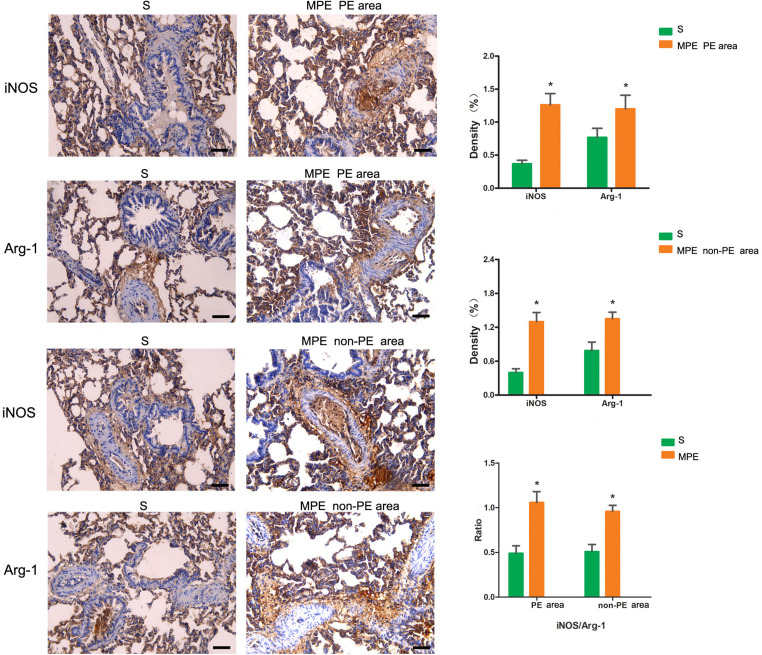
iNOS and Arg-1 immunohistochemical staining features of PE and non-PE tissues. Bar graphs showed the significant increase of iNOS, Arg-1, and iNOS/Arg-1 ratio in the MPE group (*n* = 5) compared with the S group (*n* = 4). Data are expressed as mean ± SD by bar graphs, *P < 0.05 vs. S group. PE, pulmonary embolism tissue; non-PE, non-pulmonary embolism tissue. S, sham operation group; MPE, massive pulmonary embolism group. Scale bars: 50 μm.

The expressions of iNOS and Arg-1 in pulmonary vascular and alveolar cells in PE and non-PE tissues were significantly higher in the MPE group than in the S group (*P* < 0.05, Figure 3). Meanwhile, the ratio of iNOS and Arg-1 in the PE and non-PE tissues was significantly higher in the MPE group than in the S group.

### mRNA Expression of TNF-α, IL-1β, and IL-6 in PE and Non-PE Tissues

**FIGURE 4:**
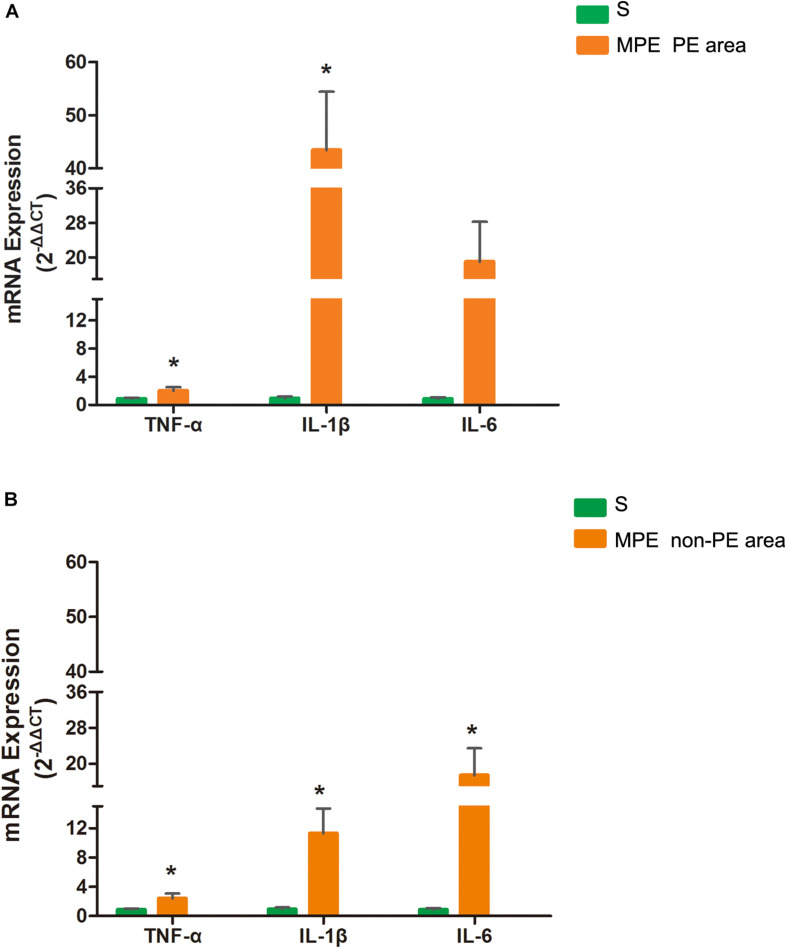
mRNA expressions of TNF-α, IL-1, and IL-6 in PE **(A)** and non-PE **(B)** tissues by RT-PCR. Bar graphs indicated that TNF-α, IL-1β, mRNA expressions of the MPE group (*n* = 5) in PE and non-PE tissues were significantly increased. IL-6 mRNA levels in non-PE tissues were significantly increased than in the S group (*n* = 4), which was tended to be higher in PE tissues. Data are expressed as mean ± SD by bar graphs. *P < 0.05 vs. S group. PE, pulmonary embolism tissue; non-PE, non-pulmonary embolism tissue. S, sham operation group; MPE, massive pulmonary embolism group.

As shown in Figure 4, mRNA expressions of TNF-α and IL-1β in PE tissues were significantly higher in the MPE group than in the S group (*P* < 0.05, Figure 4A). mRNA expression of IL-6 also tended to be higher in PE tissue in the MPE group. mRNA expressions of TNF-α, IL-1β, and IL-6 were also significantly upregulated in non-PE tissues in the MPE group than in the S group (all *P* < 0.05, Figure 4B).

### TXA2 and PGI2 Levels in PE and Non-PE Tissues

**FIGURE 5:**
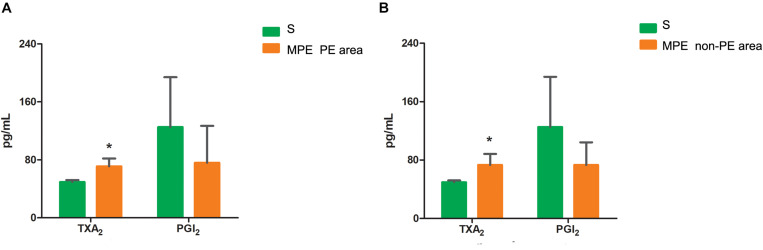
Levels of TXA_2_ and PGI_2_ in PE **(A)** and non-PE **(B)** tissues by ELISA. The vasoconstrictor factor TXA_2_ was increased and the vasodilator factor PGI_2_ of MPE group (*n* = 5) tended to be lower in PE and non-PE tissues. Data are expressed as mean ± SD by bar graphs, *P < 0.05 vs. S group (*n* = 4). PE, pulmonary embolism tissue; non-PE, non-pulmonary embolism tissue. S, sham operation group; MPE, massive pulmonary embolism group.

The TXA_2_ level was higher in both PE and non-PE tissues, while the PGI_2_ level tended to be lower in PE and non-PE tissues in the MPE group than in the S group (Figures 5A,B).

### Expression of α-SMA in PE and Non-PE Tissues

**FIGURE 6:**
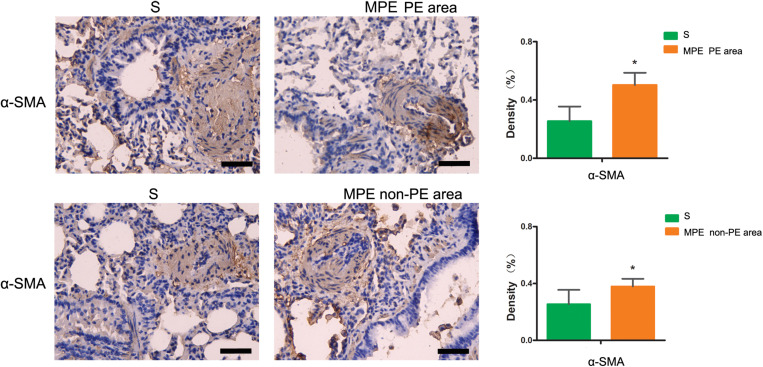
α-SMA immunohistochemical staining features of PE and non-PE tissues. The α-SMA expression in PE and non-PE tissues in the MPE group (*n* = 5) was significantly higher than in the S group (*n* = 4). Data are expressed as mean ± SD by bar graphs, *P < 0.05 vs. S group. PE, pulmonary embolism tissue; non-PE, non-pulmonary embolism tissue. S, sham operation group; MPE, massive pulmonary embolism group. Scale bars: 50 μm.

As shown in Figure 6, the α-SMA expression in PE and non-PE tissues was significantly higher in the MPE group than in the S group (*P* < 0.05).

## Discussion

Our results showed that APE induced significant inflammatory responses in this rabbit MPE model, as expressed by enhanced inflammatory cell infiltration in the pulmonary vessels in both PE and non-PE tissues; obvious edema in the interstitial cell vasculature; and increased expression of TXA_2_, TNF-α, IL-1β, and IL-6 in both PE and non-PE tissues. We believe these results might indicate a crucial role of inflammatory responses in the pathogenesis of MPE. The pathophysiological mechanisms of the high mortality caused by acute MPE combined with shock are multiple, whereas the key factor leading to shock was the acute pulmonary blood flow failure. Pulmonary artery spasm and blood flow attenuation could jointly result in the reduction of blood filling to the left ventricle. Our previous studies have shown that pulmonary artery spasm in both PE and non-PE tissues contributed to acute pulmonary failure in this MPE model (Yu et al., 2018; Wang et al., 2019). The present study further highlighted the potential role of increased inflammatory responses in both PE and non-PE tissues on enhancing the vicious progression of pulmonary hypertension in the disease course of MPE.

### The Enhanced Inflammation Response and Possible Mechanisms in Case of MPE

In this MPE combined shock rabbit model, we saw that the pulmonary vessels were filled with numerous neutrophils in both PE and non-PE tissues. Furthermore, there were significant edematous changes in vascular interstitial tissue (Figure 2). We also observed an increased expression of iNOS and Arg-1 in pulmonary vascular and alveolar cells in PE and non-PE tissues in the MPE group. It is known that iNOS is released from the vascular endothelium in response to hypoxia (Hua et al., 2018). Cheng et al. reported that the pulmonary macrophages of animal lung tissues could also secrete iNOS (Cheng et al., 2018) under pathological stimulation such as acute lung injury and inflammatory disease. The increased iNOS expression in lung tissues further indicates the enhanced inflammation status in this model. The ratio of iNOS and Arg-1 was increased in PE and non-PE tissues in the MPE group (Figure 3), indicating a hyperactive inflammation status and which might contribute to the increased expression of proinflammatory cytokines such as TNF-α, IL-1β, and IL-6 in PE and non-PE tissues of the MPE group (Figure 4). Increased inflammatory response and overexpression of inflammatory mediators may be regulated by many aspects: (1) In a previous study (Olschewski et al., 2018), the authors showed that under the stress of hypoxia, inflammatory cells such as neutrophils and macrophages could adhere and aggregate on the pulmonary artery blood vessel walls to infiltrate lung tissue. (2) The pulmonary blood flow was reduced in the setting of MPE, which could cause ischemia and hypoxia of lung tissues. Our previous study demonstrated that MAP, PaO_2_, and pH derived from blood gas analysis were significantly decreased in MPE rabbits, indicating the hypoxia status of respiratory failure in MPE rabbits associated with shock (Wang et al., 2019). Hypoxia induced generation of oxygen free radicals which are known stimulators of inflammation (He et al., 2016; Chen et al., 2020). Therefore, hypoxia might serve as a crucial factor in PE-induced inflammatory response. In addition, PE-induced pulmonary vascular spasm could also result in hypoxia and lead to a vicious circle of hypoxia-inflammation cascade in both PE and non-PE tissues in this model. (3) In our previous study, we found that the sympathetic activity of lung tissue was hyperactivated, and tyrosine hydroxylase (TH) increase could catalyze norepinephrine (NE) on vascular α-receptors; these factors could exacerbate vasospasm and simultaneously stimulate the inflammatory reaction. The above findings are in line with previous reports in that the levels of inflammatory mediators were increased in MPE and were characterized by aggregation of neutrophils and macrophages in pulmonary vasculature and reduced pulmonary blood flow (Pongratz and Straub, 2014; Barrios-Payán et al., 2016). Therefore, during the acute phase of MPE, thromboembolic vasculature, tissue ischemia, hypoxia, and the sympathetic nervous system might all mediate enhanced lung tissue inflammatory responses in this rabbit model of MPE.

### Expression of the Inflammatory Response in PE and Non-PE Tissues

The present study not only determined the inflammation of PE tissues but also evaluated the inflammation reaction in the non-PE tissues in this model. A large number of inflammatory cells infiltrated the PE tissues (Figure 2), the levels of iNOS and Arg-1 were obviously increased (Figure 3), and the mRNA expressions of pro-inflammatory cytokines including TNF-α, IL-1β, and IL-6 were all upregulated in PE tissues (Figure 4). Interestingly, non-PE tissues were also infiltrated with a large number of inflammatory cells along with obvious edema in the vascular tissues of the non-PE region. The expressions of iNOS and Arg-1 in the pulmonary vascular and alveolar tissue were significantly increased in the MPE group compared to the S group, and the ratio of iNOS/Arg-1 was significantly higher in non-PE tissues. Furthermore, the expressions of the pro-inflammatory cytokines TNF-α, IL-1β, and IL-6 were all significantly upregulated in the non-PE tissues of the MPE group compared to the S group (Figure 4B). Although the pulmonary vasculature was not blocked in the non-PE area, the pulmonary blood flow was still reduced. These phenomena might be explained as follows: (1) Under the condition of ischemia and hypoxia, a large number of neutrophils and macrophages infiltrate into the non-PE tissues and led to pulmonary vascular lesions and vascular interstitial edema, which might contribute to the pulmonary blood flow attenuation in this region. (2) The excessive activation of sympathetic activity as shown in our previous study (Wang et al., 2019) might also be an important driving force responsible for the induction of inflammatory response in non-PE areas.

### The Relationship Between Inflammation and Blood Flow Attenuation in the Whole Pulmonary Tissues

Vascular endothelial cells and smooth muscle cells play important roles in the pulmonary circulation and pulmonary blood flow. Strielkov et al. reported that hypoxia could stimulate the membrane potential on the surface of pulmonary vascular endothelial cells during the process of hypoxic pulmonary vasoconstriction, thereby contributing to the Ca^2+^ influx and pulmonary vasoconstriction (Strielkov et al., 2017). Besides hypoxia, excessive inflammation was also seen in both PE and non-PE areas post MPE, indicating a central role of inflammation in the setting of MPE besides other known factors (Trow and Taichman, 2009; Clapp and Gurung, 2015; Strielkov et al., 2017). We found that the TXA_2_ levels were obviously increased and PGI_2_ contents tended to be lower in both PE and non-PE areas (Figure 5). This finding is in line with a previous report by Foudi et al. who demonstrated that inflammatory cytokines could reduce PGI_2_ through modulating protein kinase A (PKA)/cyclic adenosine monophosphate (cAMP) pathways, and the imbalances of TXA_2_/PGI_2_ were linked with enhanced platelet aggregation, leading to vascular dysfunction in case of hypoxia (Foudi et al., 2017). Pulmonary artery smooth muscle could specifically express contraction protein α-SMA under the normal physiological condition, which played an important role in the regulation of pulmonary artery pressure (Liu et al., 2017; Li et al., 2019). Hence, we measured the expression of α-SMA in lung tissues in this model. As expected, the α-SMA expression in PE and non-PE tissues was significantly increased in the MPE group compared to the S group (Figure 6). Hypoxia, inflammatory cell infiltration in the pulmonary artery wall, vascular dysfunction, and enhanced expression of pulmonary vascular α-SMA in both PE and non-PE areas could have jointly contributed to pulmonary artery contraction and pulmonary blood flow reduction in both PE and non-PE tissues.

### Study Limitation

Our study has some limitations. First, in the setting of MPE, pulmonary artery embolization induced inflammatory response, hypoxia, pulmonary vasospasm, and blood flow attenuation; these multifaced disease features occurred in both PE and non-PE tissues. Although we evaluated inflammatory cytokines in the PE and non-PE areas in this study, we could not differentiate the exact impact of PE or hypoxia on the observed inflammatory responses in our study. Future studies are warranted to clarify this issue in the presence or absence of strategies specifically targeting the hypoxia, e.g., by supplying oxygen via a respirator to the incubated rabbit to evaluate the impact of hypoxia in this MPE model. Second, iNOS could be released from both vascular endothelium in response to hypoxia (Hua et al., 2018) and pulmonary macrophages under pathological state stimulation such as acute lung injury and inflammatory disease (Cheng et al., 2018). The present study design could not differentiate and confirm the origin of iNOS. Future studies are warranted to demonstrate the origin of iNOS by labeling pulmonary macrophages with multiple immunofluorescence tags. Thus, increased iNOS in this model could only reflect the inflammation status in our study. Third, inflammatory proteins were not measured for both mRNA and protein quantification due to experimental design deficiency. Therefore, it remains unknown whether the mRNA and protein changes were proportional; our results should thus be interpreted with caution.

## Conclusion

Excessive inflammatory response was evidenced in both PE and non-PE tissues in this rabbit model of MPE combined with shock. Inflammation might thus be one of the important factors to aggravate the pulmonary blood flow attenuation and pulmonary failure in this rabbit model of MPE combined with shock. Future studies are warranted to observe the effects of targeted intervention on inflammation in this model.

## Data Availability Statement

The raw data supporting the conclusions of this article will be made available by the authors, without undue reservation, to any qualified researcher.

## Ethics Statement

The animal study was reviewed and approved by the Animal Care Committee at Huazhong University of Science and Technology.

## Author Contributions

YG conceived and designed the study. YW, DY, and YY carried out the molecular data analyses and drafted the manuscript. YW, DY, and XL developed and supervised the animal model. LH drafted and edited the manuscript. All of the authors read and approved the final manuscript.

## Conflict of Interest

The authors declare that the research was conducted in the absence of any commercial or financial relationships that could be construed as a potential conflict of interest.

## Abbreviations

α-SMA: alpha smooth muscle actin
APE: acute pulmonary embolism
Arg-1: arginase 1
cAMP: cyclic adenosine monophosphate
ELISA: enzyme-linked immunosorbent assay
HE: hematoxylin–eosin
IL-1 β: interleukin-1 beta
IL-6: interleukin-6
iNOS: inducible nitric oxide synthase
MAP: mean arterial pressure
MPAP: mean pulmonary arterial pressure
MPE: massive pulmonary embolism
NE: norepinephrine
non-PE: non-pulmonary embolism
PE: pulmonary embolism
PGI_2_: prostaglandin I2
PKA: protein kinase A
TH: tyrosine hydroxylase
TNF- α: tumor necrosis factor-alpha
TXA_2_: thromboxane A2.
